# Gaps and Directions in Addressing Non-Communicable and Chronic Diseases in China: A Policy Analysis

**DOI:** 10.3390/ijerph19159761

**Published:** 2022-08-08

**Authors:** Yingying Jiang, Haijun Guo, Weiwei Zhang, Connie C. R. Gan, Fan Mao, Maigeng Zhou, Hai Phung, Dung Phung, Jianqun Dong, Cordia Chu

**Affiliations:** 1 Centre for Environment and Population Health, School of Medicine and Dentistry, Griffith University, Brisbane, QLD 4111, Australia; 2 National Center for Chronic and Noncommunicable Disease Control and Prevention, Chinese Center for Disease Control and Prevention, Beijing 100050, China; 3 School of Public Health, University of Queensland, Brisbane, QLD 4072, Australia

**Keywords:** policy, China, health promotion, non-communicable and chronic diseases

## Abstract

Objective: Non-communicable and chronic diseases (NCDs) have become a public health problem that seriously threatens the population’s health, especially in rapidly industrialized countries. Given the complexity of managing NCDs, there is growing evidence that interventions embedding or incorporating health promotion strategies can help reduce the disease burden of NCDs. This review aims to identify and map existing control and prevention policies for NCDs in China and identify gaps or opportunities for policy modifications and development, to transfer evidence-based guidelines into empirical public health intervention practices and research. Design: A review was conducted to evaluate the policy documents to manage NCDs in China. Keywords “chronic disease”, “health”, and “policy” were used to search documents published on the Chinese official national websites. Nvivo 12.0 was used to conduct a content analysis of the policy documents. Results: Fifty-six NCD prevention policies were retrieved from the search, and ten documents that incorporated the health promotion component were analyzed. The Healthy China 2030 Plan and Recommendations on Implementing the Healthy China Action are the core health policies of China’s Central Government. These, coupled with three nationwide community-based projects, have provided a foundational transformation platform for health promotion implementation. Conclusion: This review revealed the Chinese Government’s determination and commitment toward the prevention and control of NCDs and the promotion public health. Additional efforts and a focus on accelerating policy transformation and strengthening policy evaluation are required to achieve this commitment.

## 1. Introduction

Over the past 30 years, the global epidemic of non-communicable and chronic diseases (NCDs) has brought an increasingly heavy disease burden on the general public. The high and increasing prevalence of NCDs is a significant health problem faced by developed countries and poses an even more formidable challenge for resource-limited countries, with 85% of premature NCD deaths [1,2]. China’s disease burden has changed dramatically over the past three decades, with 86% of deaths resulting from NCDs [3]. To achieve the United Nations Sustainable Development Goals (SDGs) of reducing premature death by 30%, the prevention and control of NCDs will play an essential role [4].

Conventional NCD prevention focuses on lifestyle behavior, such as diet and physical activity. However, more evidence shows that natural and social environments profoundly impact an individual’s behavior. In China, with the development of the social economy, the health challenges faced by the population are increasingly closely related to lifestyle and social determinants, such as urbanization and population aging [5,6,7]. From a broader and social perspective, a comprehensive strategy needs to be adopted for the prevention and control of NCDs [8,9]. Studies in industrialized countries show that preventing NCDs and their risk factors through health promotion strategies can produce more substantial benefits for maintaining public health than clinical treatment measures [10,11]. Health promotion enables people to increase control and improve their health, broaden the traditional considerations on health, and address prerequisites for health such as economic resources, a stable ecosystem, and sustainable development. Despite its importance, little is known about how health promotion was incorporated into NCDs policies in China. Some studies also argued the lack of comprehensive and cross-sectoral policies for China’s chronic disease prevention and control [12,13].

We compiled this review to identify and describe existing policies targeting the four types of NCDs control and prevention based on the Ottawa Charter action plan. We identify gaps/opportunities for policy modifications to incorporate health promotion components in NCDs policies. Such an analysis aims to support the current understanding of China to address NCDs with more alignment towards achieving SDG goals.

## 2. Methods

### 2.1. Design

A document review was conducted to evaluate the policy documents to prevent NCDs in China. The deadline for policy searches is July 2022.

### 2.2. Data Sources

According to the World Health Organization, health policy is defined as “decisions, plans, and actions that are undertaken to achieve specific health care goals within a society” (https://www.who.int/topics/health_policy/en/, accessed on 28 June 2021). Keywords of “chronic and non-communicable disease(s)”, “non-communicable disease(s)”, “chronic disease(s)”, “health”, or “policy” were applied to search documents published on the official websites of the State Council and the National Health Commission of the People’s Republic of China.

### 2.3. Data Analysis

A thematic analysis approach using concept- and data-driven coding was used for the study. Before the independent document review, the authors agreed on the inclusion and exclusion criteria. All types of written materials published in Chinese included a focus on health promotion to control four major NCDs (cardiovascular diseases, cancers, chronic respiratory conditions, and type 2 diabetes) and their risk factors (tobacco use, physical inactivity, the harmful use of alcohol, and unhealthy diets). The inclusion criteria comprised: official documents or policies issued by the Central Government of China and documents or policies, including plans, recommendations, guides, and outlines, published in Chinese. Exclusion criteria included: documents or policies not related to NCDs; documents, such as notices, pictures, or protocols; duplicated documents; expired documents as of 2022; and documents targeted at the subnational level or a specific province in China.

First, two reviewers (the primary and the secondary authors), applied the inclusion and exclusion criteria to the identified documents. Titles of policy documents were reviewed, and complete documents were read after the reviewers agreed on the relevance of documents based on their titles. Then, two authors assessed the full documents separately and reached a consensus on the documents to be included for analysis. After this, each document was critically read to identify key concepts (codes). The key concepts identified were then categorized according to their represented ideas. These categories were then analyzed and classified according to predetermined themes from the policy analysis work. In this review, the Ottawa Charter framework, which contains five actions, was applied as the code for the content analysis of the included policies [9,14]. Only policies that contain all five actions of the Ottawa Charter—developing personal skills, reorienting health services, strengthening community action, creating supportive environments, and building healthy public policy—were included in the thematic analysis.

The authors reviewed data extraction forms developed in Microsoft Excel. Data were extracted by the principal author and audited by the second author. Key elements of the data extraction included organization, year of policy issue, goals, objectives, strategies, and actions of the policies. Nvivo 12.0 (QSR International, Doncaster, Australia) was applied to conduct the content analysis of the policy documents.

Consultations with experts and stakeholders were conducted several times during the review process. Feedback was collected from health authority officials in the Central Government of China, preventive medicine, and public health experts in universities (Griffith University and domestic universities in China).

## 3. Results

### 3.1. Overview of the Policy Documents

**Figure 1:**
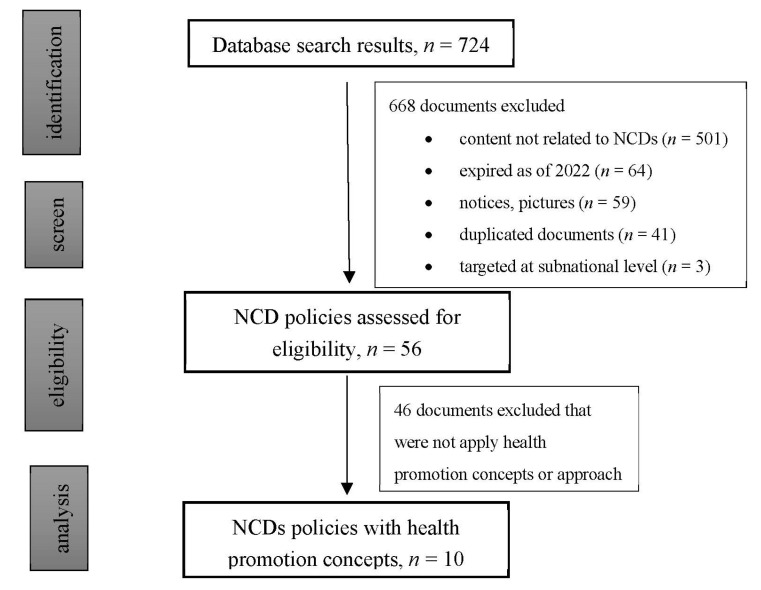
Flow diagram of policy documents identification and search.

**Figure 2:**
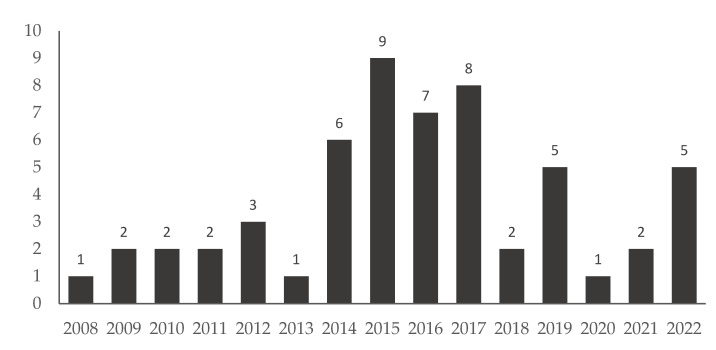
The number and issued year of NCD prevention policies in China.

The search retrieved a total of 724 policy documents, including 104 documents published by the State Council of China and 620 documents published by the National Health Commission, China. Documents not targeting the four major types of NCD or NCD-related risk factors were excluded (N = 668). The remaining 56 NCD policies were issued between the years 2008 and 2022. Most documents were published between 2014 and 2017. Ten documents were included for thematic analysis based on the health promotion framework (see Figure 1 and Figure 2 for details), and all ten of these documents were issued after 2015. Key elements of the ten documents are summarized in Table 1.

### 3.2. Policies Addressing NCDs in China

The Healthy China 2030 Plan and Recommendations on Implementing the Healthy China Action are China’s essential health policies at the Central Government level. In 2016, China’s State Council issued the Healthy China 2030 Plan, a grand blueprint to promote a healthy China and implement major initiatives promised in the 2030 Agenda for Sustainable Development. At the core of the Healthy China 2030 Plan are four principles: health priority, innovation, scientific development, and fairness and justice. Strategies applied in this plan include a people’s health center; taking reform and innovation as the driving force and prevention as the primary strategy; integrating health into all policies; targeting health-related risk factors, such as behavior, production, and living environments; targeting medical and health services; adhering to a combination of Government leadership and the mobilization of society and individuals; encouraging community participation; and strengthening early diagnosis, early treatment, and early recovery. This plan proposes thirteen goals, of which three goals, closely related to the prevention and control of NCDs, were to reduce the premature mortality of major NCDs (30% reduction in 2030 compared with 19.1% in 2015), to improve residents’ health literacy level (reaching 30% in 2030), and to increase the number of residents regularly participating in physical exercise (530 million in 2030).

In 2019, the State Council issued Recommendations on Implementing the Healthy China Action, which was supposed to facilitate the implementation of the Healthy China 2030 Plan. This document proposed 15 specific actions related to the prevention and control of chronic diseases, including (a) healthy diet action, (b) national fitness action, (c) tobacco control action, (d) health environment promotion action, (e) cardiovascular and cerebrovascular disease prevention action, (f) cancer prevention action, (g) COPD prevention and control action, and (h) diabetes prevention action. Detailed targets have been proposed in several particular actions for the prevention and control of major NCDs, such as the decline in the mortality from cardiovascular and cerebrovascular diseases to 190.7/100,000 or lower in 2030, the five-year survival rate of overall cancer no lower than 46.6%, a decrease in COPD mortality rate to 8.1/100,000 or lower, and the standardized management rate of diabetes patients to reach 70% and above. The introduction of Recommendations on Implementing the Healthy China Action has increased the transformability of the Healthy China 2030 Plan.

The National Health Commission, the National Development and Reform Commission, the Ministry of Education, the Ministry of Ecology and the Environment, the Ministry of Finance, and ten other ministries or commissions in China jointly launched the Health China Action-Cancer Prevention and Control Implementation Plan (2019–2022). This plan outlines the overall goal of curbing the rise in cancer morbidity and mortality, with the hope of increasing the overall five-year survival rate of cancer by 3% from 2015 to 2022. This plan advocates corresponding actions in eight areas, including: health promotion, vaccination, water quality improvement, soil and atmospheric environment, regulation of tumor patient registration, tumor screening and early diagnosis, and standardized treatment.

To address the physical activity promotion issue, which is one of the four risk factors of NCDs, it is proposed in the National Fitness Plan (2021–2025) that, by 2025, the public service system for national fitness will be substantially improved, people’s physical fitness will be more conveniently achieved, the enthusiasm for fitness will be further improved, the number of participants in various sports projects will continue to increase, and the proportion of people who regularly participate in physical exercise will reach 38.5%. Community 15 min fitness circles will be established to achieve full coverage, with 2.16 social sports instructors per 1000 people. The plan also proposes promoting the development of national fitness in three aspects: deepening the integration of sports and education, promoting the integration of sports and health, and promoting the integration of sports and tourism. It is necessary to explore the establishment of an exercise-promoting health model in which sports, health, and other departments are involved. Moreover, integrated exercise and health services are planned to be delivered at the community level.

### 3.3. Implemented Initiatives and Projects to Achieve the Blueprint

Three nationwide initiatives and projects have been implemented to realize the blueprint of Healthy China 2030. These projects are the Patriotic Health Campaign project, the Construction of the National Demonstration Areas for Non-communicable Diseases Comprehensive Prevention and Control project (NDAP), and the National Healthy Lifestyle Action project.

Opinions on Deepening the Patriotic Health Campaign and Recommendations of the State Council on Further Strengthening Patriotic Health Campaign in the New Period are focused on the governance of environmental sanitation and improving population health literacy. Management Measures on the Construction of the National Demonstration Areas for Noncommunicable Diseases Comprehensive Prevention and Control puts forward straightforward management methods for several demonstration areas built at the district/county level, requiring the implementation of Health in All Policies (HiAP), the construction of a supportive environment, the integrated system for NCDs prevention and treatment, health education and health promotion, and carrying out work on NCDs management. National Healthy Lifestyle Action Plan (2017–2025) is a policy document jointly issued by the National Health and Family Planning Commission, the General Administration of Sports, the National Federation of Trade Unions, the Communist Youth League, and the Women’s Federation. This document advocates the development of NCD-related risk factors control, such as diet, exercise, tobacco control, the reduction in harmful alcohol consumption, mental health projects, and improving the population’s health literacy.

### 3.4. Policies Focusing on the Governance of Health Care System Development and Planning

The 14th Five-Year National Health Plan and China’s Medium- and Long-Term Plan for the Prevention and Treatment of Chronic Diseases (2017–2025) are two health care system development and reform policies. One of the missions of the 14th Five-Year National Health Plan is to implement a comprehensive prevention and control strategy for NCDs. Key points in this policy include improving the Government-led comprehensive prevention and control mechanism, establishing a community-based NCDs prevention and control system, and strengthening the implementation of NDAP with a coverage of 20% in the counties (cities, districts) in 2025. China’s Medium- and Long-Term Plan for the Prevention and Treatment of Chronic Diseases (2017–2025) is under the framework of the Healthy China 2030 Plan and is mainly applied in the field of NCDs prevention and control. The policy sets 16 specific work goals, focusing on four significant NCDs and three behavioral risk factors (tobacco use, high level of salt intake, and low physical activity). This document advocates projects in four areas: health promotion projects, health management projects, supportive environmental construction projects, and NCDs science and technology research projects. It also proposes to increase the coverage of the NDAP to 20% by 2025.

## 4. Discussion

The presented review highlights the key policies addressing NCDs prevention and control and population health promotion from 2015 to the present. Ten policies were searched on NCDs control and prevention by incorporating a health promotion strategy. Four blueprints, three projects, and two health care system development and planning strategies were described and analyzed.

### 4.1. NCD Policy Development Aligned with Sustainable Development

These blueprints policies have emerged after 2016 and are evolving towards a new stage of China’s health promotion efforts. Unlike the previous disease-oriented health policy, these health policies attempt to embody a people-centered concept, actively advocate prevention-oriented strategies, and adopt health promotion concepts to maintain the population’s health. In the 21st century, health is increasingly regarded as an essential determinant of sustainable development, and policies and government commitments are crucial for maintaining the population’s health. OECD and G20 countries have made political commitments to safeguard the health of their citizens [15,16]. The United Nations developed the SDGs based on the successful implementation of the Millennial Development Goals. Goal 3, “Ensuring healthy lives and promoting well-being at all ages is essential to sustainable development”, revolves around health issues. Avoiding 30% premature deaths of NCDs by 2030 is one of the tasks advocated by SDGs [17]. Although China has made notable achievements in maternal and child health care and infectious disease prevention and control, addressing the NCD problem is still a considerable challenge [18]. The launch of the Healthy China 2030 Plan outlines the overall vision, goals, and tasks for the construction of a healthy China from a strategic and overall perspective and proposes principles for health work. The Recommendations on Implementing the Healthy China Action further proposed 15 particular actions, such as health knowledge popularization action, healthy environment promotion action, women’s and children’s health promotion action, which increase the operability of the Healthy China 2030 Plan. These blueprint policies have contributed to a favorable policy environment for China to achieve SDGs.

### 4.2. Health Promotion Action Plan to Implement NCD Policies

The health promotion action plans, first proposed in the Ottawa Charter in 1986 and reiterated and improved in the 2016 Shanghai Declaration, are proven to help promote and protect public health [9,19,20]. The health promotion policy advocates population-based work and focuses on cultivating community capabilities or empowerment [21,22]. Three important health promotion projects exist in districts, counties, and cities in China, including the Patriotic Health Campaign, NDAP, and the National Healthy Lifestyle Action project.

China’s Patriotic Health Campaign started in 1950 and is a population-based health movement with a long history. The Patriotic Health Campaign initially aimed t improve hygiene conditions and reduce vector-borne diseases. In 2014, the WHO fully realized the achievements of the Patriotic Health Campaign in China [23]. The Patriotic Health Campaign has a well-organized operation system and a mass foundation in China and will play an essential role in addressing NCDs. The concept of health promotion was subsequently applied to China’s planned immunization and AIDS prevention and control work. It was not until the end of the last century, with the support of the World Health Organization, that the pilot work for the comprehensive prevention and the control of chronic diseases carried out in China began to adopt health promotion strategies in the field of chronic disease prevention and control [24]. In 2010, the Ministry of Health of China expanded this pilot work and launched the NDAP [25]. Local governments at the district or county level was required to guide the establishment of the demonstration area with the participation of the multisector. The prevention and treatment of NCD have been combined, and both individual risk factors and social determinants of NCD have been targeted, covering 488 counties and districts in 31 provinces across mainland China. The NDAP has been characterized by mobilizing government departments and advocating the concept of HiAP [26]. The National Healthy Lifestyle Action started in 2007, and its core features are attention to behavioral risk factors for NCDs, such as smoking, alcohol drinking, excessive salt intake, lack of exercise, and active advocacy to create a healthy and supportive environment [27]. The operation and implementation of these national health promotion projects provide a good platform for transforming the blueprint policy.

## 5. Areas for Improvement

Based on the findings of this review, we have also discovered some policy gaps in the application of health promotion strategies to prevent and control chronic diseases in China. One of the notable gaps in China’s policies is the failure to address cardiovascular disease, COPD, and diabetes with a health promotion strategy. Only the Cancer Prevention and Control Implementation Plan has been issued with a health promotion approach. No specifically designated and separately released policy documents exist to prevent and treat the other three major NCDs. Our findings also show that China currently only has policies for health promotion related to physical activity, while other policies for the three major risk factors associated with NCDs (unhealthy diet, smoking, and harmful drinking) have not yet been introduced. Hence, we propose that existing national policies and actions on risk factors be reinforced with health promotion strategies.

## 6. Limitations of the Analysis

The scope of this review was limited to Central Government policies and relies on the available official policies as the primary source of data analysis. Therefore, peer-reviewed policy analysis articles were not included. The analyzed policies are limited to documents issued by the Central Government of China. Local governments in China develop policies based on the Central Government’s policies, which are also aligned with national legislation. While national policies contain overarching goals, principles, and a roadmap, local policies focus on priorities, resource allocation, and implementation processes. Consequently, the findings of this review cannot reflect China’s regional differences and features.

## 7. Conclusions

This paper provided information for advocating and improving policy development to address NCDs in China. In recent years, the Chinese Government has attached increasing importance to the comprehensive prevention and control of NCD by issuing health promotion and comprehensive prevention and control policies. China has already issued blueprint policies that conform to the principles of health promotion (Healthy China 2030 Plan and Recommendations on Implementing the Healthy China Action). Currently, China has established three community-based projects covering the whole country (the Patriotic Health Campaign, the NDAP, and the National Healthy Lifestyle Action), which have provided a solid ground for transforming health promotion policies. Implementation research improves the adoption of these health policies and accelerates policy action, which is a critical priority in the next step. The timely identification, assessment, and problem-solving implementation will facilitate the fulfillment and realization of the Healthy China 2030 Plan Goals. To facilitate policy implementation and translation, we recommend strengthening policy evaluation, including annual monitoring, as well as mid-term and final assessment of the policy planning and implementation.

## Figures and Tables

**Table 1:** Overview of the policies for NCD control and prevention developed with the health promotion concept.

No.	Title of the Policy	Published Year	Published Department
1.	Recommendations of the State Council on Further Strengthening Patriotic Health Campaign in the New Period	2015	State Council, China
2.	Healthy China 2030 Plan	2016	State Council, China
3.	Management Measures on the Construction of the National Demonstration Areas for Noncommunicable Diseases Comprehensive Prevention and Control	2016	National Health Commission, China
4.	China’s Medium- and Long-Term Plan for the Prevention and Treatment of Chronic Diseases (2017–2025)	2017	State Council, China
5.	National Healthy Lifestyle Action Plan (2017–2025)	2017	National Health Commission, China
6.	Recommendations on Implementing the Healthy China Action	2019	State Council, China
7.	Health China Action-Cancer Prevention and Control Implementation Plan (2019–2022)	2019	National Health Commission, China
8.	Opinions on Deepening the Patriotic Health Campaign	2020	State Council, China
9.	National Fitness Plan (2021–2025)	2021	State Council, China
10.	14th Five-Year National Health Plan	2022	State Council, China

## Data Availability

Not applicable.
